# Astrocytic CD24 Protects Neuron from Recombinant High-Mobility Group Box 1 Protein(rHMGB1)-Elicited Neuronal Injury

**DOI:** 10.3390/brainsci12091119

**Published:** 2022-08-23

**Authors:** Cong Pang, Sen Gao, Xun-Zhi Liu, Xiao-Jian Li, Zheng Peng, Hua-Sheng Zhang, Yan Zhou, Xiang-Xin Chen, Tao Tao, Yue Lu, Wei Li, Chun-Hua Hang

**Affiliations:** 1 Department of Neurosurgery, Nanjing Drum Tower Hospital, The Affiliated Hospital of Nanjing University Medical School, Nanjing 210008, China; 2 Department of Neurosurgery, Nanjing Drum Tower Hospital, Clinical College of Nanjing Medical University, Nanjing 210008, China

**Keywords:** HMGB1, CD24, astrocytes, inflammatory response

## Abstract

Endogenous host-derived molecules named damage-associated molecular patterns (DAMPs) can induce excessive non-sterile inflammatory responses on recognition of specific membrane-tethered receptors. Here in this study, we aimed to explore the role of DAMP molecule HMGB1 in astrocyte-mediated sterile neuroinflammation and the resultant influences on neurons. In vitro cultured astrocytes were challenged with rHMGB1 and then harvested at 6 h, 12 h, 24 h, 36 h, and 48 h, respectively. The astrocytic CD24 expression was determined by quantitative real-time polymerase chain reaction (qPCR), Western blot analysis and immunofluorescence, nuclear factor kappa B (NF-κB) binding activity was detected by electrophoretic mobility shift assay (EMSA), and the proinflammatory factors, tumor necrosis factor-α (TNF-α), and interleukin 1β (IL-1β), were measured by qPCR. The neuronal morphology was assessed with phase-contrast microscopy. The results showed that astrocytic mRNA and protein CD24 expression began to rise at 24 h, peaked at 36 h, and remained elevated at 48 h after rHMGB1 stimulation, accompanied with enhanced NF-κB binding activity and augmented expression of TNF-α and IL-1β. Furthermore, rHMGB1 caused cocultured neuron damage and was aggregated upon CD24 knockdown. Taken together, these novel findings suggested that rHMGB1 could promote astrocytic CD24 expression, the inhibition of which could aggregate neuronal damage.

## 1. Introduction

Excessive inflammation is thought to be closely related with many central nervous system (CNS) injuries [1]. Upon receiving the initial damage, the injured cells would release a large amount of damage-associated molecular patterns (DAMPs) molecules, causing a cascade of non-sterile inflammatory reactions [2]. Though an appropriate degree of inflammation is beneficial for the removal of harmful substances [3,4], the body usually exhibits an excessive inflammatory response as a result of continuous stimuli exposure, such as the unresolved hematoma clearance in cerebral hemorrhage [5]. Such long-term stimulation can lead to a poor clinical prognosis. The high mobility group box 1 (HMGB1) is such a DAMP, identified as an actively secreted cytokine produced by inflammatory cells or a medium released passively from injured cells [6]. Following brain injury, the released HMGB1 binds to its receptors and mediates specific signaling pathways promoting inflammation, the resultant upregulation of TNF-α, IL-1β, and other cytokines could finally cause damage to the CNS [7,8].

So far, the inhibition of excessive inflammation has been widely regarded as a feasible solution to rescue CNS injury. On these grounds, many researchers focus on microglia, given its role as inherent CNS immune cells. Regretfully, they have encountered failure in transforming these studies into clinical utilization [9]. Therefore, emerging studies have shifted interest towards astrocytes [10]. The astrocytes, which account for the majority of central cells, are responsible for the maintenance of the CNS structure and embedded with various functions including cytokine secreting, were neglected [11,12,13]. Although it has been long suggested that the astrocytes also play a role in the inflammatory response [14,15], there is still a lack of research on its specific mechanism. Therefore, it is worthwhile to investigate how the astrocytes participate the neuroinflammatory process.

On binding with specific receptors, DAMPs can initiate certain feedbacks [16], and studies have pointed out that CD24, a membrane receptor also known as heat stable antigen (HSA) in mouse, implements such function [17,18]. The evidence has shown that CD24 can discriminate DAMPs from the pathogen-associated molecular patterns (PAMPs) [19]. Besides, the interaction of CD24 and Siglec-10 reduces the DAMPs-associated inflammation by inhibiting the activation of HMGB1 and nuclear factor kappa B (NF-κB) [19,20,21,22]. Our previous research has also found that CD24 can enhance neuronal regeneration in experimental subarachnoid hemorrhages [23]. Therefore, we speculate that the astrocytes might modulate inflammation by interacting with the DAMPs through membrane-tethered alarmin CD24. To verify this hypothesis, we designed this study to investigate the expression of CD24 in the rHMGB1-stimulated astrocytes and its impact on the cocultured neurons, and tried to find out the potential mechanisms of CD24 in modulating sterile inflammations.

## 2. Materials and Methods

### 2.1. Primary Cell Culture

For the primary neurons, timed-pregnant female C57BL/6 mice were euthanized, and 14 to 16 embryos were collected by cesarean section. The cortex was isolated and rinsed with precooling phosphate-buffered saline (PBS) and treated with TrypLE Express Enzyme (Cat No. 12604021; Thermo Fisher Scientific Inc., Grand Island, NE, USA) for 10 min at 37 °C, and then the cells were triturated with heat-polished glass pipettes and filtered by a 40-μm-diameter strainer (Cat No. CLS431750; Corning, Glendale, AZ, USA). After centrifugation, the cells were resuspended in Neurobasal media (Cat No. 21103049; GIBCO, Life Technologies, Grand Island, NE, USA) supplemented with B27 (Cat No. 17504044; GIBCO, Life Technologies, USA) and were dissociated by repeated pipetting through a 1 mL blue Eppendorf pipette tip. Then, the cells were seeded at 1.5 × 10^6^ cells per well in 6-well poly-D-lysine (Cat No. A-003-M; Sigma Chemical Co., Darmstadt, Germany)-precoated plates. The cultures were maintained at 37 °C in a humidified atmosphere of 5% CO_2_ and 95% air. Similar procedures were completed for the primary astrocytes; the astrocytes were resuspended and cultured in Dulbecco’s modified eagle medium (DMEM) basal media (Cat NO. 11965092; GIBCO, Life Technologies, USA) supplemented with 10% fetal bovine sera (Cat NO. 10100147; GIBCO, Life Technologies, USA) and penicillin–streptomycin (Cat NO. C100C5; New Cell & Molecular Biotech Co., Suzhou, China). Subsequently, the medium was replaced every 2 days; 48 h prior to coculture, the astrocytes were transferred to poly-D-lysine precoated Transwell inserts (Cat NO. CLS3396, pore size = 0.4 μm; Corning, NY, USA) and cultured until 100% confluence. An indirect astrocyte–Neuron coculture system was then established with the neurons seeded in the lower chamber and the astrocytes in the upper Transwell chamber for 24 h.

### 2.2. rHMGB1 Stimulated Sterile Inflammation Model

The recombinant HMGB1 (Cat NO. SRP2051; Sigma Chemical Co., St. Louis, MO, USA) was diluted in DMEM and applied to the astrocytes culture at day 10 after plating, and the cells in the rHMGB1 groups were stimulated for 6 h, 12 h, 24 h, 36 h, and 48 h, respectively. According to the relevant research, the final concentration of rHMGB1 was set at 100 ng/mL. Meanwhile, the DMEM without rHMGB1 was applied to the astrocytes in the control group.

### 2.3. RNA Isolation and Quantitative Real-Time PCR

The in- vitro cultured cells were rinsed twice with PBS. The total RNA was collected from each group (*n* = 6), with the use of TRIZOL Reagent (Cat NO. 740955.50; TAKARA Biotechnology, Dalian, China), following the manufacturer’s recommendations. The reverse transcription was carried out using Reverse Transcriptase Kit (TAKARA Biotechnology, Dalian, China) under the manufacturer’s recommendations. The forward and reverse primers used for the cDNA amplification were as follows: 5′-GTTGCTGCTTCTGGCACTG-3′, 5′-GGTAGCGTTACTTGGATTTGG-3′ for CD24; 5′-TCTCATTCCTGCTTGTGGC-3′, 5′-CACTTGGTGGTTTGCTACG-3′ for TNF-α; 5′-GGGCTGCTTCCAAACCT-3′, 5′-GATGTGCTGCTGCGAGA-3′ for IL-1β; 5′-GACAGGATGCAGAAGGAGATTACT-3′, 5′-TGATCCACATCTGCTGGAAGGT-3′ for β-actin, respectively, and were synthesized from Life Technologies (Invitrogen, Shanghai, China). The amplification and data acquisition were run on a PCR system (Applied Biosystems, Woburn, MA, USA), applying real-time SYBR Green Master Mix (Roche, Shanghai, China) adopting 2^−ΔΔCt^ method. All of the procedures were performed according to the Minimum Information for publication of Quantitative real-time PCR Experiments Guidelines (The MIQE Guidelines).

### 2.4. Total/Cytosolic/Nuclear Protein Extraction

Total protein extraction: The cultured cells were rinsed twice with PBS and each well was homogenized in 200 μL ice-cold RIPA buffer (Cat No. 89901; Thermo Scientific, MA, USA) supplemented with 1% protease and phosphatase inhibitor cocktail (Cat NO. P1005; Beyotime, Shanghai, China) and centrifuged at 12,000× *g* for 15 min at 4 °C. The protein concentrations were estimated by commercial Protein Assay Reagent kit (Cat NO. P0012; Beyotime, Shanghai, China). The total protein extracts were collected and stored at −80 °C before Western blot analysis.

The cytosolic/nuclear protein extraction was conducted under product instructions: the cultured cells were rinsed twice with PBS and each well was homogenized in 200 μL ice-cold buffer A with 1 mM PMSF (Cat NO. ST505; Beyotime, Shanghai, China). The homogenate was vortexed and incubated on ice for 15 min. After 10 μL Buffer B supplementation, the mixture was vortexed for 5 s and incubated on ice for 1 min and then centrifuged for 15,000× *g* for 5 min. The cytosolic supernatant was transferred to another tube. Then, 50 μL nuclear extraction buffer was added to the sediment, vortexed for 20 s and incubated on ice every 1 min, this procedure was repeated for 30 min and then centrifuged at 14,000× *g* for 15 min at 4 °C. The supernatant was transferred to another tube.

### 2.5. Western Blot Analysis

Equal amounts (40 μg per lane) of protein were loaded and separated by SDS-polyacrylamide gel electrophoresis. Then, the protein was transferred to a polyvinylidene difluoride membrane (Cat NO. HVLP04700; Millipore, Billerica, MA, USA). Following blockage of the non-specific binding site using Quick-block kit (Cat NO. P0252; Beyotime, Shanghai, China) for 15 min at room temperature, the primary antibody against CD24 (Cat NO. ab64064; Abcam, Cambridge, UK) or β-actin (Cat NO. BS6007MH; Bioworld Technology, Louis Park, MN, USA) was added and incubated overnight. Then, the protein-loaded membrane was washed using 1 × tris-buffered saline with Tween 20 (TBST) for 3 × 5 min and incubated with horseradish peroxidase (HRP)–conjugated secondary antibody (ab7097; Abcam, Cambridge, UK) at room temperature for 2 h. The protein bands were visualized, adapting Enhanced Chemiluminescent HRP substrate Ultra Reagent (Cat NO. P2200; New Cell & Molecular Biotechnology, Suzhou, China).

### 2.6. Immunofluorescence

The treated astrocytes were fixed in 4% pure formaldehyde at 4 °C overnight, blocked with 5% goat serum in PBST, and incubated with primary anti-CD24 antibody (Cat NO. ab64064; Abcam, Cambridge, UK) for 2 h at 37 °C. After 3 × 5 min washes with PBS, the astrocytes were then incubated at 37 °C for 40 min, utilizing Alexa Fluor 594-conjugated Goat Anti-Rabbit IgG (Cat NO. ab 7074; diluted 1:200, Invitrogen, Shanghai, China) in darkness. The results were obtained with a fluorescence microscope (LSM 880; Zeiss, Heidenheim, Germany).

### 2.7. RNA Interference

The lentiviral knockdown of CD24 was conducted as previously described [24]. Briefly, CD24-specific shRNA or vehicle were added to 6-well plates at a dose of 10^9^ per milliliter. The astrocytes were used in subsequent experiments 72 h after transfection. The Western blot analysis and quantitative real-time PCR were used in the evaluation of the knockdown efficiency.

### 2.8. Electrophoretic Mobility Shift Assay (EMSA)

EMSA was performed using a commercial assay kit (Cat NO. E3300, Gel Shift Assay System; Promega, Madison, WI, USA) to evaluate the NF-κB DNA-binding activity as previously reported. Briefly, the HeLa nuclear extract was used as a positive control. The NF-κB probe(5′-AGTTGAGGGGACTTTCCCAGGC-3′) was then mixed with [γ^−32^P] ATP (Cat No. GS056; Beyotime, Shanghai, China) and T4-polynucleotide kinase (Cat NO. D7098L; Beyotime, Shanghai, China). The binding mixture was then added to the nuclear extract (40 μg protein) and incubated at 37 °C for 10 min. Then, the reaction mixture was subjected to electrophoretic separation on 4% native polyacrylamide gel in 0.5 × TBE buffer. Following the transmembrane procedure, visualization was conducted using HRP-labeled Streptavidin (Cat NO. A0303; Beyotime, Shanghai, China).

### 2.9. Terminal Deoxynucleotidyl Transferase-Mediated dUTP Nick and Labeling (TUNEL)

The TUNEL staining of the cultured neuron was performed, in accordance with the manufacturer’s instructions (Cat No. C1901; Beyotime, Shanghai, China). Briefly, the cells were incubated with a primary antibody against NeuN (Cat NO. 26975; Proteintech, Wuhan, China) overnight and then incubated with secondary antibody. The cells were then incubated with biotin-conjugated Terminal Deoxynucleotidyl Transferase for 30 min at room temperature and visualized under laser microscope. The NeuN^+^/TUNEL^+^ cells were deemed as the apoptotic neurons.

### 2.10. Statistical Analysis

All of the data were presented as mean ± SD. GraphPad Prism 7.0 was used for the statistical analysis of the data. The measurements were subjected to one-way analysis of variance (ANOVA) followed by Tukey’s post-hoc test. A value of *p* < 0.05 was considered statistically significant.

## 3. Results

### 3.1. Expression and Distribution of CD24 in Cultured Astrocytes after rHMGB1 Stimulated

**Figure 1:**
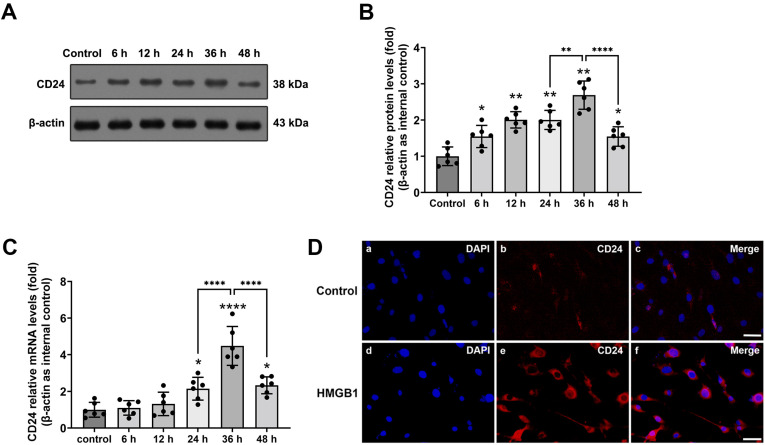
Expression and distribution of CD24 in cultured astrocytes after rHMGB1 stimulated. (**A**) Protein expression of CD24 in astrocytes using β-actin as loading control. Lane 1, control; lane 2, 3, 4, 5, and 6, the 6 h, 12 h, 24 h, 36 h, and 48 h groups, respectively; (**B**) Graphic representations of the ratios of CD24/β-actin protein. Bars represent the mean ± SD (*n* = 6, each group); (**C**) The mRNA expression of CD24 in cultured astrocytes measured by quantitative real-time PCR. Bars represent the mean ± SD (*n* = 6, each group). * *p* < 0.05, ** *p* < 0.01, **** *p* < 0.0001 vs. control or indicated groups; (**D**) Immunofluorescence staining for CD24 in cultured astrocytes. (**a**–**c**) CD24 expression in the control group (DAPI = blue, CD24 = red); (**d**–**f**) CD24 expression in the rHMGB1 group at 36 h (DAPI = blue, CD24 = red). Bar = 50 μm. Statistical comparison was conducted using one-way ANOVA; *p* < 0.05 was considered statistically significant.

The expression of the CD24 protein was determined by Western Blot (Figure 1A,B) (see Figure S1A, Supplementary Materials). A low level of CD24 protein was found in the control group, while in the 24 h, 36 h, and 48 h groups, the CD24 expression significantly increased. There was a significant statistical difference between the control group and the 24 h, 36 h, and 48 h groups (*p* < 0.01, respectively).

The expression of the CD24 RNA was determined by real-time PCR (Figure 1C). An extremely low level of CD24 mRNA was found in the control group, while significantly increased mRNA expression of CD24 in the cultured astrocytes was detected in the 24 h, 36 h, and 48 h groups (24 h, 36 h: *p* < 0.01; 48 h: *p* < 0.05). The results showed that the CD24 mRNA expression increased sharply by 24 h, peaked at 36 h, and remained elevated at 48 h after the rHMGB1 application in the cultured astrocytes.

Besides, the expression and localization of CD24 were assessed by immunofluorescence at the peak activation of CD24 (36 h after rHMGB1 application, according to real-time PCR and Western blot). In the control group, a weak expression of CD24 was observed in the cultured astrocytes, while the increased CD24-positive astrocytes could be found in the 36 h group, following application of rHMGB1 (Figure 1D) (see Figure S1B, Supplementary Materials).

### 3.2. NF-κB DNA-Binding Activity in Cultured Astrocytes after rHMGB1 Stimulation

**Figure 2:**
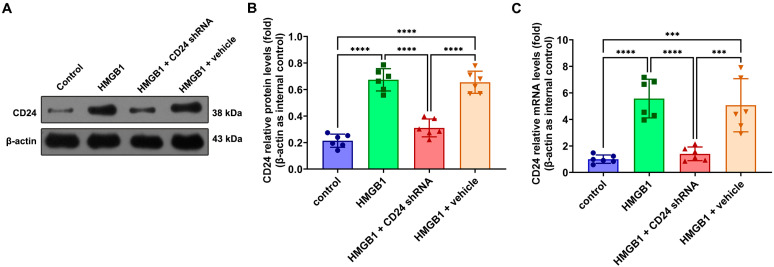
Expression of CD24 decreased after shRNA interference. (**A**) Protein expression of CD24 in astrocytes using β-actin as loading control; (**B**) Graphic representations of the ratios of CD24/β-actin protein; (**C**) mRNA expression of CD24 in astrocytes using β-actin as loading control. Bars represent the mean ± SD (*n* = 6, each group). *** *p* < 0.001, **** *p* < 0.0001 vs. control or indicated groups. Statistical comparison was conducted using one-way ANOVA; *p* < 0.05 was considered statistically significant.

To test the impact of the CD24 on the astrocytes, we used shRNA for silencing and evaluated the silencing effect. Both the Western blot and real-time PCR showed that the application of shRNA reduced the expression of CD24 in the astrocytes to an extremely low level (Figure 2) (see Figure S2, Supplementary Materials).

**Figure 3:**
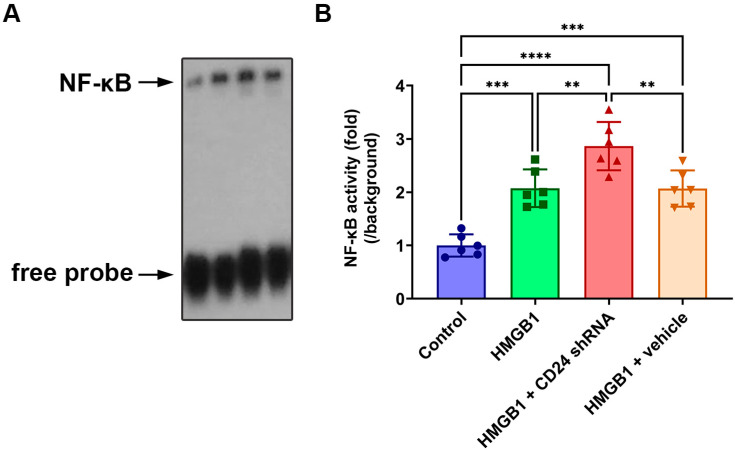
EMSA was used to explore the NF-κB DNA-binding activity in cultured astrocytes in all groups. (**A**) Measurement of NF-κB DNA-binding activity in cultured astrocytes in all groups. Lanes 1, 2, 3, and 4 represent the control group, HMGB1 group, HMGB1 + shRNA group, and HMGB1 + NC group, respectively; (**B**) Quantitative analysis showed that NF-κB DNA-binding activity in HMGB1 + shRNA group was markedly higher than any other. Bars represent the mean ± SD (*n* = 6, each group). ** *p* < 0.01, *** *p* < 0.001, **** *p* < 0.0001 vs. control or indicated groups. Statistical comparison was conducted using one-way ANOVA; *p* < 0.05 was considered statistically significant.

Subsequently, the EMSA autoradiography of the NF-κB DNA-binding activity in the cultured astrocytes following rHMGB1 stimulated is shown in Figure 3. A low NF-κB binding activity (weak EMSA autoradiography) was found in the control group, while in the HMGB1-challenged groups, the NF-κB DNA binding activity was progressively upregulated with the time from 6 h to 48 h and peaked at 24 h after the rHMGB1 stimulation. As compared with that in the control group, the NF-κB DNA binding activity in each HMGB1 stimulation group showed a significant statistical difference (*p* < 0.01, respectively).

### 3.3. The Morphology Damage of Cocultured Neurons Deteriorated after the Knockdown of CD24 in Astrocytes

**Figure 4:**
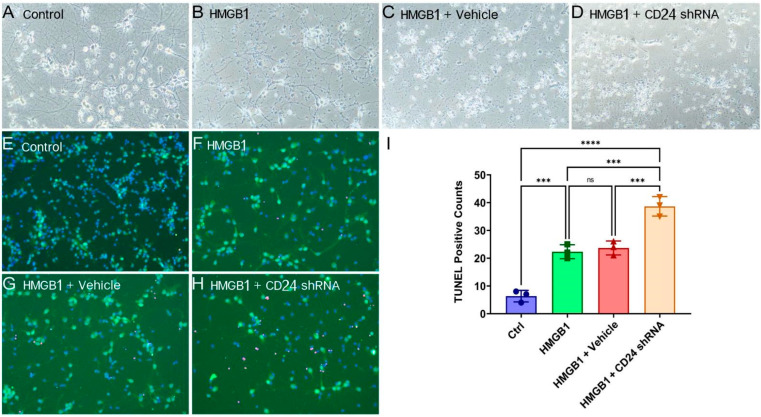
Representative photomicrographs of cocultured neurons under light microscopy (**A**–**D**). Representative photomicrographs (**E**–**H**) and quantification (**I**) of TUNEL immunofluorescence staining. Green stains for NeuN, red stains for TUNEL, and DAPI stains for nucleus. Scale bar: 20 μm. Ns = not significant, *** *p* < 0.001, **** *p* < 0.0001 vs. control or indicated groups. Statistical comparison was conducted using one-way ANOVA; *p* < 0.05 was considered statistically significant.

The morphology of the cocultured neurons was observed under the phase-contrast light microscope and fluorescence microscope (Figure 4). The co-cultured neurons showed destruction in the axons, cell soma shrinkage and apoptosis in the HMGB1 group compared with the control group, and the damage worsened in the HMGB1 + shRNA group, with a minimum number of axons, a maximum degree of cell body shrinkage and apoptosis.

### 3.4. TNF-α and IL-1β mRNA Expression Levels in Cultured Astrocytes after rHMGB1 Stimulation

**Figure 5:**
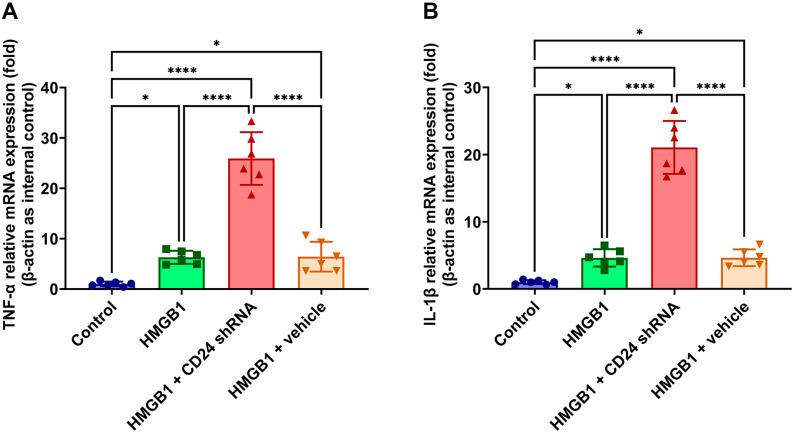
The mRNA expression of TNF-α and IL-1β in cultured astrocytes were measured by quantitative real-time PCR. mRNA levels of TNF-α in HMGB1 group were higher than that in the control group and lower than that in HMGB1 + shRNA group (**A**), mRNA levels of IL-1β in HMGB1 + shRNA group were higher than that in the control group and lower than that in HMGB1 + shRNA group **(B**), Bars represent the mean ± SD (*n* = 6, each group). * *p* < 0.05, **** *p* < 0.0001 vs. control or indicated groups.

According to the real-time PCR results, the TNF-α and IL-1β mRNA levels remained at a low-level in the control group. Compared with those in the control group, the TNF-α and IL-1β mRNA levels both increased significantly in the HMGB1 group (*p* < 0.01, respectively) (Figure 5). The results also suggested that the TNF-α and IL-1β mRNA expressions in the cultured astrocytes increased at 6 h, peaked at 12 h, and remained elevated at 48 h after application of rHMGB1.

## 4. Discussion

**Figure 6:**
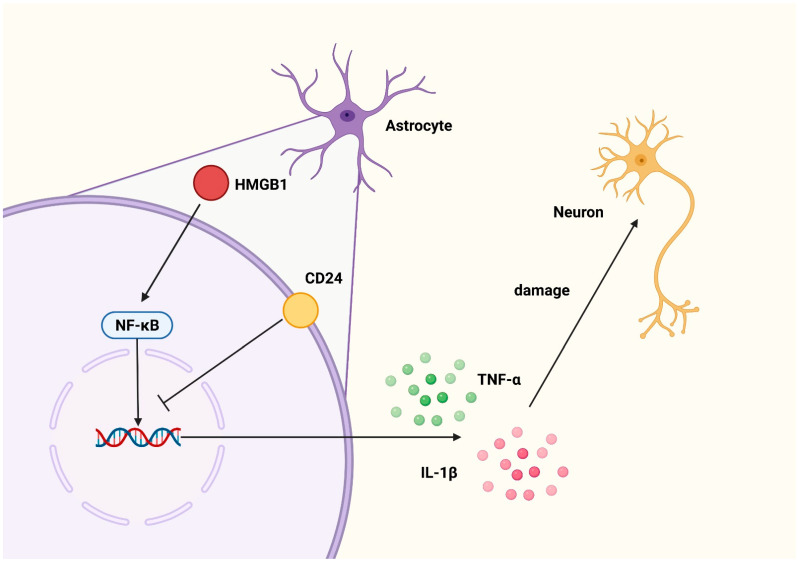
Graphical abstract of the present research. The zoom on the left shows that astrocytes, after being stimulated by HMGB1, will activate the NF-κB pathway and cause the release of downstream inflammatory factors, such as TNF-α and IL-1β, thus causing damage to neurons. Meanwhile, CD24 can play an inhibitory role in this process by inhibiting the DNA binding ability of NF-κB. Figure was created via BioRender.com (accessed on 17 December 2021).

In this present study, we observed the inflammatory activation of the astrocytes following the rHMGB1 challenge. The results showed that the astrocytic CD24 expression was promptly upregulated following rHMGB1 stimulus, most notably at 36 h; additionally, the NF-ΚB pathway was activated. Furthermore, the proinflammatory cytokines TNF-α and IL-1β expression increased, and the neurons were co-cultured. Interestingly, when the expression of CD24 is silenced, the upregulation of the NF-ΚB and inflammatory factors will be more significant, the CD24 expression repression further augmented the upregulation of NF-κB and associated inflammatory factors and aggregated neuronal injury in the co-cultured neurons. As shown in Figure 6, these results suggest that CD24 may be involved in inhibiting the HMGB1-induced astrocytic activation of NF-ΚB and downstream inflammatory pathways, thereby promoting the survival of the neurons.

These findings may contribute to resolving many of the neuroinflammation-associated neurological diseases, especially those with acute courses, such as stroke. A classic pathological process of these diseases is DAMPs, molecules released by damaged cells or tissues, which cause successive proinflammatory activations, such as the microglia or astrocytes [25]. Subsequently, when the cascade of inflammation exceeds the normal range, the neurons will be injured and lead to an undesirable clinical prognosis [26].

As one classic DAMP molecule, HMGB1 is a highly conserved protein that can be released by injured or dying cells [8,27]. A growing body of evidence has proved that HMGB1 could be elevated in the settings of sepsis, shock, and other inflammatory diseases, if the strategies to reduce HMGB1 activity have a therapeutical effect that has not yet been validated [28]. There are two major releasing patterns for HMGB1 during injury, either active or passive [29,30,31]. The active release of HMGB1, commonly initiated by cellular signal transduction through plasma membrane-tethered receptors via an interaction with various extracellular ligands, occurs slowly. Meanwhile the passive release is mostly initiated after cellular integrity disturbance, which occurs nearly instantaneously. The extracellular HMGB1 is known to be able to induce complex cascades of signaling via binding to certain receptors, including RAGE, TLR2, and TLR4 [32,33,34]. Afterwards, HMGB1 can trigger inflammation mediated by well-conserved pathways, such as mitogen-activated protein kinase (MAPK), NF-κB, and activator protein 1 transcriptional responses [35,36]. Moreover, increasing evidence has found that HMGB1 can promote its own release in vivo and in vitro in a feedforward manner, and the astrocytes can be stimulated to actively secrete HMGB1 [37]. Based on these findings, in the present study, we utilized rHMGB1 to stimulate the cultured astrocytes to simulate the instantaneous release (passive release); as a result, it induced the active release of HMGB1 in the astrocytes, thereby cascading the sterile inflammatory response and finally inducing a DAMPs-dependent CNS injury model in vitro.

Given the fact that astroglia comprises the majority of the cellular masses of the brain and the current research on microglia has seen limited therapeutic effect, we embarked on the study of the astrocytes [10,38,39]. As major component, the astrocytes provide structural support and necessary nutrients for the neurons, while uncontrollable astrocytic reaction might secrete excessive inflammatory factors [10,38,39]. As for how the astrocytes respond to the HMGB1 stimulant, our results indicated that CD24 might participate in repressing the HMGB1-induced inflammatory process. The studies indicated that CD24 can be used to recognize DAMPs [19], and the CD24-Siglec-G/10 pathway may have the function of decreasing the host’s response to DAMPs by the selective repression of NF-κB DNA binding activity [39]. Therefore, we propose a hypothesis that CD24 might also have its own immunomodulatory functions. To date, no study in the literature has focused on the role of CD24 alone in the in vitro simulated inflammatory experiments. In our present study, we demonstrated for the first time that CD24 was activated in the cultured astrocytes after rHMGB1 application and partially blocked the damage of the cocultured neurons.

Additionally, further investigation of the CD24 working mechanism has found that NF-κB, one of the classic proinflammatory nuclear factors, might take effect downstream of the CD24 signaling. Our results showed that CD24 expression escalated sharply as NF-κB binding activity peaked, and remained elevated as NF-κB binding activity decrease; given the temporal alterations of these two molecules, we further performed a further knockdown of the astrocyte CD24 expression, which aggregated the NF-κB binding activity and proinflammatory factors’ secretion, causing more severe neuronal damage, confirming that CD24 might act as a negative regulator in repressing the HMGB-induced and NF-κB mediated astrocytic proinflammatory process. This illustrates that CD24 can react as a negative immune-sensing alarmin to inhibit the HMGB1/NF-κB pathway-mediated inflammation.

In summary, we found that the astrocytes could ameliorate the neuronal damage via the CD24-mediated inhibition of the NF-κB binding activity and subsequent inflammatory factors’ secretion following the HMGB1 challenge. Former studies have investigated the astrocytic function in maintaining normal central nervous function, owing to its massive quantities and various functions [40]. Recently, astrocytes were proved to phagocytose the neuronal debris [41,42] or modulate neuronal functions via neurotransmitter recycling, ion homeostasis, or metabolic coupling [41,43,44], while the astrocyte–neuron immune interaction remains elusive. In this study, we observed elevated astrocytic CD24 expression following the rHMGB1 challenge, and the shRNA silencing of the astrocytic CD24 expression could aggregate the neuron morphology damage in the neuron–astrocyte co-culture system, suggesting that the astrocytes could partially, if not entirely, protect the neurons from the rHMGB1-induced astrocyte-mediated inflammation and ameliorate synapse damage; therefore, our results have illustrated the existence of an immune interaction between the neurons and astrocytes apart from the previous astrocytic structural scaffold and nutrient-supporting functions. In the realm of brain injury, the microglia are thought to be the first to react, and the microglia-mediated long-term unresolved reactive inflammation could cause an undesirable prognosis [45], while the question as to how and to what extent the astrocytes participate in such a pathological process and the corresponding impacts on the neurons warrants further exploration.

## 5. Conclusions

In summary, we found that the astrocytes may reduce neuronal damage through the CD24-mediated inhibition of NF-κB binding activity and the subsequent release of downstream inflammatory factors following HMGB1 stimulation. These results should be more accurately validated in future studies.

## Data Availability

The data presented in this study are available on request from the corresponding author.

## Supplementary Materials

The following supporting information can be downloaded at: https://www.mdpi.com/article/10.3390/brainsci12091119/s1, Western blot and immunofluorescence staining support Figure S1, Figure S2.
