# Supplementary material for: Astrocytic CD24 Protects Neuron from Recombinant High-Mobility Group Box 1 Protein(rHMGB1)-Elicited Neuronal Injury
Source: Brain Sci. 2022 Aug 23;12(9):1119. doi: 10.3390/brainsci12091119 (PMC9497078; doi:10.3390/brainsci12091119)

Supplementary Materials:

Figure S1.

Figure S1A: full blots support Figure 1A:

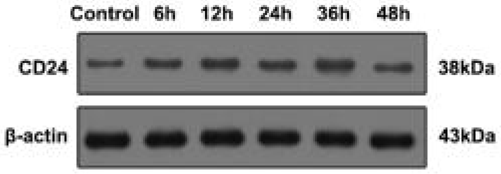

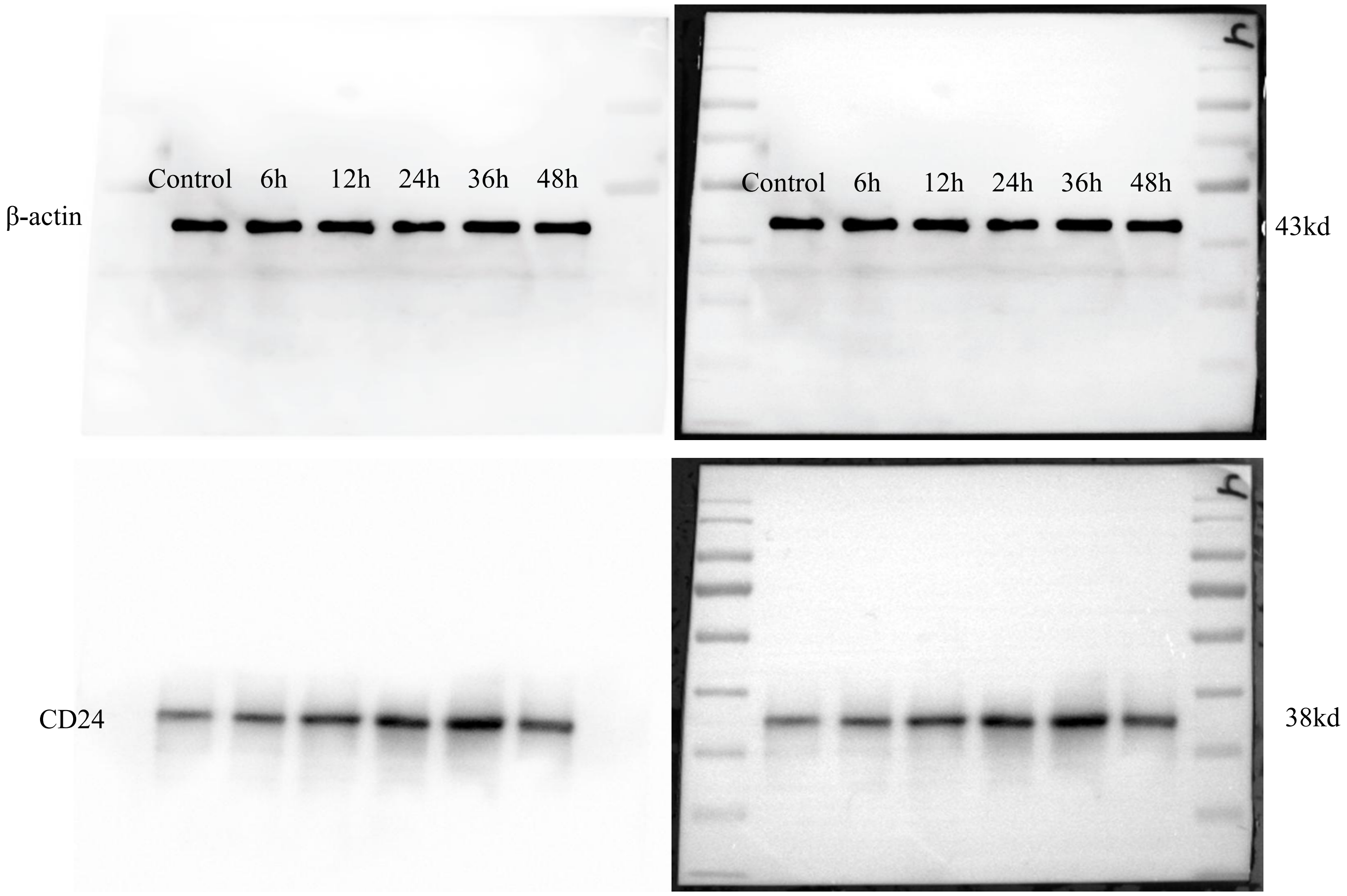

Figure S1B: cell type identity support Figure 1D:

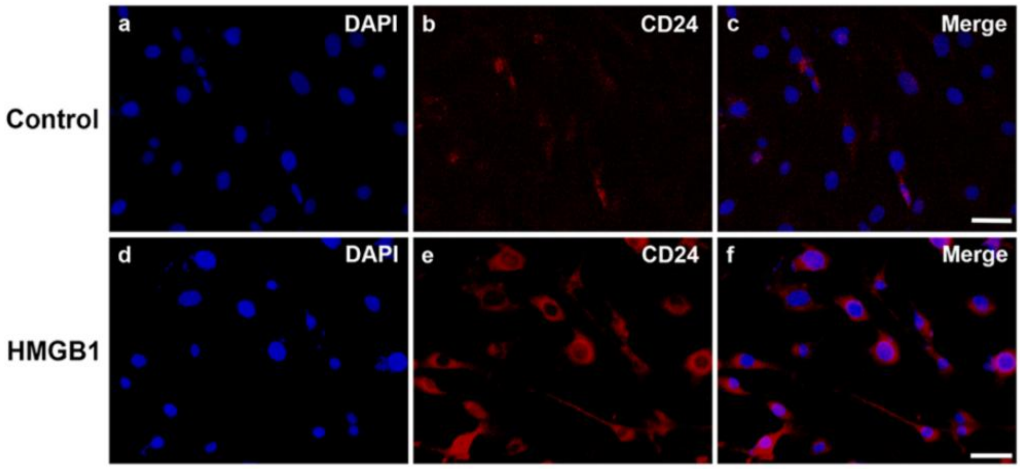

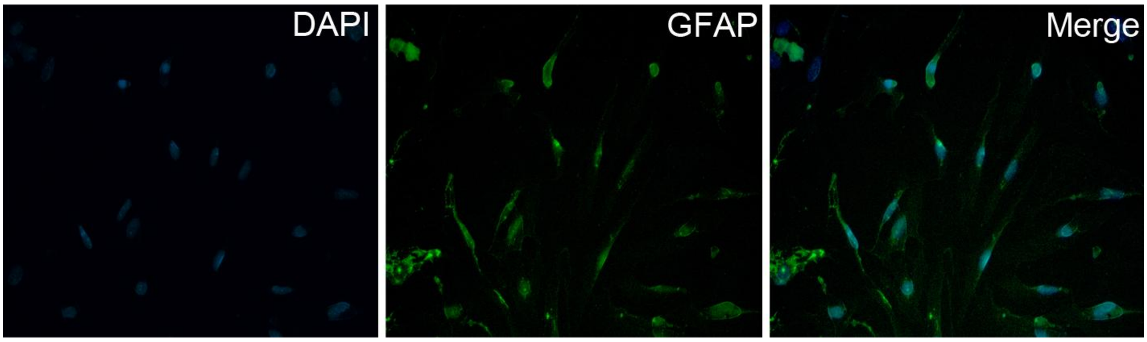

Figure S2: full blots support Figure 2A

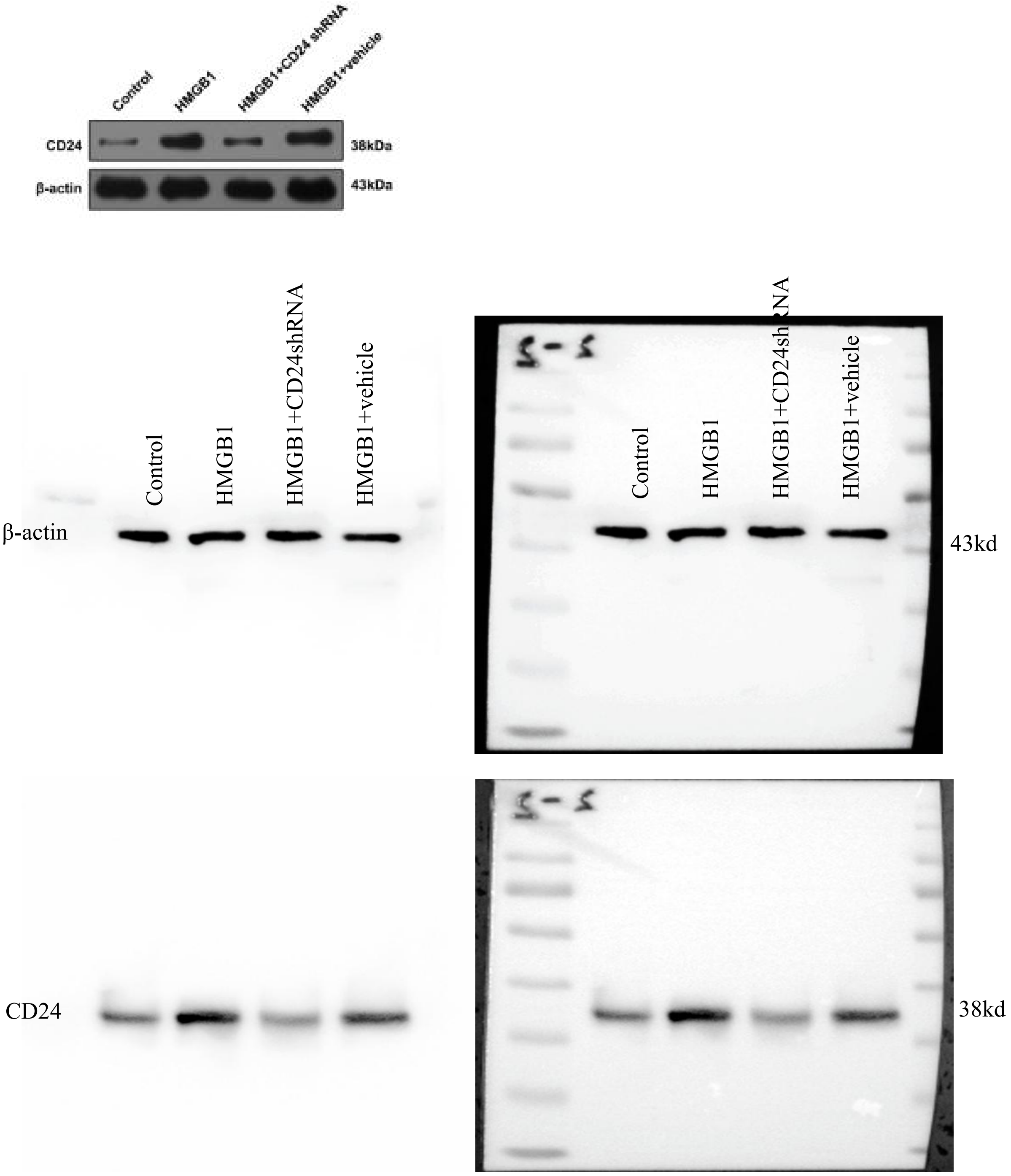

Supplement: Supplementary file 1 [file brainsci-12-01119-s001.zip › brainsci-1776416-supplementary.pdf]
